# Sex Differences in Vagus Nerve Stimulation Effects on Rat Cardiovascular and Immune Systems

**DOI:** 10.3389/fnins.2020.560668

**Published:** 2020-11-06

**Authors:** Farid Yaghouby, Kee Jang, Uyen Hoang, Sepideh Asgari, Srikanth Vasudevan

**Affiliations:** U.S. Food and Drug Administration, Center for Devices and Radiological Health (CDRH), Office of Science and Engineering Laboratory (OSEL), Division of Biomedical Physics (DBP), Silver Spring, MD, United States

**Keywords:** sex differences, vagus nerve stimulation, cardiovascular system, immune system, neuromodulation

## Abstract

**Background:**

Investigations into the benefits of vagus nerve stimulation (VNS) through pre-clinical and clinical research have led to promising findings for treating several disorders. Despite proven effectiveness of VNS on conditions such as epilepsy and depression, understanding of off-target effects and contributing factors such as sex differences can be beneficial to optimize therapy design.

**New Methods:**

In this article, we assessed longitudinal effects of VNS on cardiovascular and immune systems, and studied potential sex differences using a rat model of long-term VNS. Rats were implanted with cuff electrodes around the left cervical vagus nerve for VNS, and wireless physiological monitoring devices for continuous monitoring of cardiovascular system using electrocardiogram (ECG) signals. ECG morphology and heart rate variability (HRV) features were extracted to assess cardiovascular changes resulting from VNS in short-term and long-term timescales. We also assessed VNS effects on expression of inflammatory cytokines in blood during the course of the experiment. Statistical analysis was performed to compare results between Treatment and Sham groups, and between male and female animals from Treatment and Sham groups.

**Results:**

Considerable differences between male and female rats in cardiovascular effects of VNS were observed in multiple cardiovascular features. However, the effects seemed to be transient with approximately 1-h recovery after VNS. While short-term cardiovascular effects were mainly observed in male rats, females in general showed more significant long-term effects even after VNS stopped. We did not observe notable changes or sex differences in systemic cytokine levels resulting from VNS.

**Comparison With Existing Methods:**

Compared to existing methods, our study design incorporated wireless physiological monitoring and systemic blood cytokine level analysis, along with long-term VNS experiments in unanesthetized rats to study sex differences.

**Conclusion:**

The contribution of sex differences for long-term VNS off-target effects on cardiovascular and immune systems was assessed using awake behaving rats. Although VNS did not change the concentration of inflammatory biomarkers in systemic circulation for male and female rats, we observed significant differences in cardiovascular effects of VNS characterized using ECG morphology and HRV analyses.

## Introduction

The vagus nerve innervates multiple organs and plays a critical role in a number hemostatic and health-promoting physiologic pathways (Groves and Brown, 2005). Onset and progression of several diseases are known to be linked with autonomic dysfunction, particularly suppression in vagal tone (Koopman et al., 2011). Hence, electrical activation of this nerve, vagus nerve stimulation (VNS) or VNS, has been explored to provide therapeutic benefits (Yuan and Silberstein, 2016). VNS, previously approved by the U.S. Food and Drug Administration (FDA) for treatment of epilepsy and depression (FDA, 1997), has been recently cleared for treatment of migraine and cluster headache (Yuan and Silberstein, 2016; FDA, 2018; Lendvai et al., 2018). In recent years, VNS has gained attention to treat cardiovascular conditions (e.g., heart failure), neurological disorders (e.g., tinnitus and stroke), and chronic inflammatory diseases (e.g., rheumatoid arthritis, Crohn’s disease and sepsis), with limited considerations about personalized study design and inadequate understanding on therapeutic paradigms (Ben-Menachem et al., 2015).

VNS signaling pathways for cardiac and inflammatory functions have been extensively documented in the literature. The sensory vagal pathway, incorporating afferent nerve bundles from mainly the left cervical vagus nerve, communicates with the nucleus tractus solitarius (NTS) in the brainstem via cardiac and immunological signals (Tracey, 2009). The therapeutic mechanism of VNS particularly in epilepsy is known to be through this sensory vagal pathway (Henry, 2002). To minimize adverse cardiac effects in clinical practice, the left cervical vagus nerve has been preferentially stimulated over the right cervical branch (Yuan and Silberstein, 2016). The right branch primarily innervates the sinoatrial (SA) node which can directly alter the heart rate, whereas the left branch innervates the atrioventricular (AV) node which has less influence on the frequency of heart beats (Ardell and Randall, 1986). The immunomodulatory function of the vagus nerve through the cholinergic anti-inflammatory pathway (CAP) has been first demonstrated in VNS studies using animal models (Pavlov and Tracey, 2005). Through this pathway, afferent and efferent nerve fibers can activate or inhibit the release of pro-inflammatory cytokines, respectively (Pavlov and Tracey, 2005). VNS has been shown to regulate pro-inflammatory cytokine release by activating the descending cholinergic efferent pathway to potentially treat inflammatory diseases (Tracey, 2002; Hoover, 2017). Stimulation of CAP via VNS may suppress the serum levels of pro-inflammatory cytokines such as TNF-α and also limit production of such proteins in the corresponding organs such as liver and spleen (Tracey, 2002).

Despite demonstrated VNS effectiveness to suppress seizure yield in epilepsy and improve the mood in depression patients, a variety of side effects including hoarseness, coughing, dyspnea, and respiratory complications during sleep have been reported by patients (Fahy, 2010). Since the heart is mainly innervated by parasympathetic inputs via the vagus nerve, cardiac symptoms such as bradycardia, bradyarrhythmia, and changes in heart rate variability (HRV) have also been observed during intraoperative implant testing and long-term use of VNS, although with substantial inter-subject variability (Frei and Osorio, 2001; Ardesch et al., 2007). Several studies have indicated that autonomic regulation of cardiovascular function reflected as HRV can be subtly impaired by VNS, but without substantial and everlasting effects on cardiovascular modulation (Stemper et al., 2008; Cadeddu et al., 2010). The alterations in heart rate dynamics through VNS have been investigated in patients with drug-resistant epilepsy and reported as overall suppression in HRV (particularly sympathovagal balance) and improvement in impaired heart rhythm complexity reflected as linear and non-linear analyses (Liu et al., 2017, 2018). To assess the safety of VNS in non-disease conditions, acute VNS (using left cervical nerve) on control dogs did not reveal any significant effects on cardiac autonomic balance assessed by HRV analyses (Martle et al., 2014).

Clinical evidence for sex differences in cardiovascular function and dynamics have been well documented in healthy subjects. A comprehensive literature review on potential sex differences in HRV features from healthy human subjects has revealed that females show significantly lower RR interval, higher variability (measured as standard deviation of RR intervals or SDNN), and greater vagal activity shown as lower LF/HF power ratio (i.e., elevated high-frequency [HF] and reduced low-frequency [LF] components for RR interval power spectral analysis) than men (Koenig and Thayer, 2016). However, there are limited studies investigating the impact of biological sex on VNS-induced cardiac effects reflected by HRV. A recent investigation on the correlation between morphology of the cervical vagus nerve and parasympathetic activity characterized by HRV in healthy subjects has revealed that there is a significant correlation between morphology of the left cervical nerve and the autonomic regulation of the cardiovascular function, where among different demographic data including gender, only age was a significant cofactor (Pelz et al., 2019). On VNS side effects, in a small cohort of epilepsy patients treated with VNS, the proportion of female subjects with respiratory side effects during exercise was significantly higher than that of males, where four out of total five patients with respiratory side effects were female (Mulders et al., 2015). Although effects of sex difference in neuroimmune response and release of inflammatory cytokines have been extensively studied (Pascual et al., 2017; Osborne et al., 2018), potential differences in VNS therapy were only found in one study where remission by VNS seemed to be less effective in a female-only cohort (Koopman et al., 2016).

Considering anatomical differences between male and female in sympathetic and parasympathetic innervation of internal organs including heart and spleen (Groves and Brown, 2005), the effects of VNS and potential sex differences on autonomic regulation of cardiovascular and immune systems have not yet been studied. Investigation on potential sex differences in off-target effects of VNS may shed light into our understanding about associated risks for this therapy. Also, it may enhance VNS benefits by considering sex differences in the design and implementation. Hence, there is an unmet need to explore different aspects of VNS off-target effects on cardiovascular and immune functions considering long-term use and sex differences. The purpose of the present study was to determine if VNS can alter cardiovascular parameters and plasma cytokine levels in control rats and whether these changes indicate any potential sex differences. This has been achieved using rodent models of chronic VNS recently developed by our group (Yaghouby et al., 2019). Male and female rats were implanted with wireless electrocardiogram (ECG) recording devices and cuff electrodes around the left cervical vagus nerve. After recovery from surgeries, VNS was performed for eight weeks using a wireless programmable pulse generator in awake behaving animals without the use of anesthesia. ECG signals were recorded continuously and analyzed for assessment of cardiovascular system effects. Weekly blood samples were analyzed to characterize the expression of inflammatory biomarkers to study VNS effects on the immune system. Our results showed significant sex differences mainly in cardiovascular effects of VNS, which points to the importance of biological sex differences as a critical factor for developing therapeutic applications using VNS.

## Materials and Methods

### Animal Protocol and Experimental Design

**FIGURE 1:**
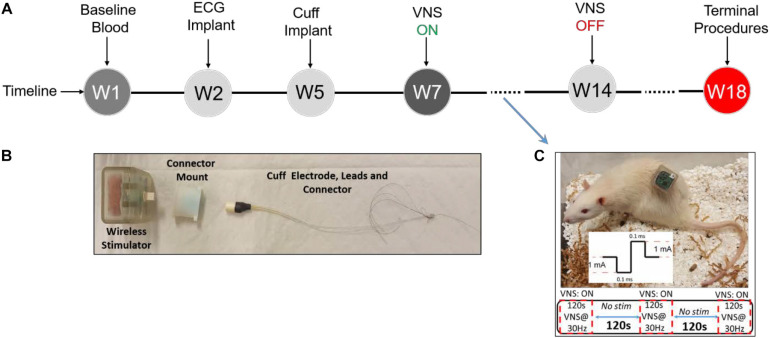
Experimental design. **(A)** Experimental timeline. **(B)** Vagus nerve cuff electrode, connector mount (prototype), and wireless stimulator. **(C)** Wireless stimulator connected to an awake behaving rat during experiment, along with VNS parameters.

Animal experiments were performed in accordance with the National Research Council Guide for the Care and Use of Laboratory Animals and approved by the FDA’s Institutional Animal Care and Use Committee (IACUC). Male (*n* = 8, 250–340 g) and female (*n* = 8, 204–223 g) Lewis rats were purchased at the age of 12 weeks and evenly randomized to Treatment or Sham groups (four males and four females in each group). A rat model for long-term VNS experiments with wireless physiological monitoring was used in this study. Surgical procedures, electrode design, and initial validation of this animal model have been thoroughly explained elsewhere (Yaghouby et al., 2019). In summary, after the acclimatization period, as shown in Figure 1A, each rat underwent two successive implantation surgeries, one for the wireless ECG recording device (EMKA Technologies, Inc.; Paris, France) and another for the vagus nerve cuff electrode (Microprobes for Life Sciences; MD, United States) and the transcutaneous connector mount for VNS (Figures 1B,C). Two weeks after implantation of the cuff electrode at week 5 (W5), VNS was turned on and continued for eight weeks (W7–14) in awake behaving animals from the Treatment group, based on parameters shown in Figure 1C. Rats from the Sham group were implanted with both ECG recording devices and cuff electrodes and monitored similarly but did not receive VNS. Continuous wireless ECG recordings and weekly blood draws were performed from all animals (Treatment and Sham) from W1 onward.

### Electrode and Stimulator Design

As shown in Figure 1B, our electrode design consists of two components: a bipolar nerve cuff electrode (Microprobes for Life Sciences; MD, United States) and a transcutaneous connector mount. The cuff electrode wires were connected to a Micro 360 Plastic Circular connector (MCS-05-SS, Omnetics Connector Corporation; MN, United States) and enclosed in a custom 3D-printed connector mount. Details on electrode and connector mount design and fabrication were previously explained (Yaghouby et al., 2019). The difference for this study is that we did not use magnets for our connector mounts and instead secured the stimulator device using male Omnetics connectors (MCP-05-SS, Omnetics Connector Corporation; MN, United States). A wireless programmable pulse generator (Triangle Bioscience International-TBSI; NC, United States) was modified to fit our needs of this study. The stimulator was encapsulated in a 3D-printed plastic casing and equipped with a male connector for convenient interface with the cuff electrode connector (Figure 1B). The wireless pulse generator was paired with a PC using StimWare software (Triangle Bioscience International – TBSI; NC, United States) for programming and automated delivery of the stimulus with desired parameters. The wireless pulse generator was equipped with a rechargeable battery to be disconnected and charged between VNS sessions.

### Surgical Procedures and Study Protocol

Details on implantation of ECG monitoring device (at W2) and cuff electrode and connector mount (at W5) were thoroughly explained in our previous publication (Yaghouby et al., 2019). Briefly, to implant the ECG device, an incision was made in anesthetized rats to create a small opening into the peritoneal cavity to insert the device, and leads were sutured to the chest muscle to record telemetric ECG. After a 3-week recovery from the first surgery, rats were implanted with VNS cuff electrodes around the left cervical vagus nerve and a connector mount over the lumbar fascia to interface a wireless pulse generator. Aseptic techniques were used to make a ventral midline incision in the neck to access and isolate the left cervical vagus nerve from the carotid artery sheath. A custom 1-mm-diameter bipolar cuff electrode (Microprobes for Life Science; MD, United States) was implanted around the nerve. The cuff electrode leads were then tunneled through the neck and connected to a connector mount secured by sutures to the animal’s lumbar fascia. A wireless constant-current pulse generator was connected to the mount to interface with the cuff electrodes during each VNS session. At the end of the study (W18), rats were euthanized followed by device explanation. Appropriate lead placement and long-term functionality of the used rat model have been previously confirmed by intermittent impedance measurement and explant procedures (Yaghouby et al., 2019). Identical surgical procedures, signal acquisition, and blood sampling were carried out for the Sham group except that Sham rats did not receive VNS.

### Data Collection

#### Blood Draw and Cytokine Analysis

Once a week, approximately 1 mL of blood was collected from the tail vein in each rat. During blood collection, rats were anesthetized with 3% isoflurane. A twenty-one-gauge butterfly needle tip was inserted into a lateral tail vein, and the blood was collected into an anticoagulant EDTA-coated tube (Fisher Scientific; NH, United States). The tube was gently inverted several times to mix blood with the anticoagulant and transferred into an Eppendorf tube stored on ice until centrifugation at a speed of 3,000 rpm for 10 min. Blood plasma was then harvested and stored at −80°C for further processing. The levels of inflammatory cytokines in the blood plasma were analyzed by rat enzyme-linked immunosorbent assay (ELISA) for TNF-α (SRTA00, R&D Systems; MN, United States), IL-1β (SRLB00, R&D Systems), IL-6 (SR6000B, R&D Systems), and IFN-γ (SRIF00, R&D Systems) according to the manufacturer’s protocols. The plates were read on a spectrophotometric plate reader (Spectra Max M5, Molecular Devices; Sunnyvale, CA, United States). The cytokine levels before surgeries (W1) represented as baseline.

#### ECG Recording and VNS Protocol

Electrocardiogram signals were continuously recorded at 500 Hz sampling frequency from each rat throughout the course of the study starting from implantation of the wireless ECG device at W2. Telemetric ECG data from 16 rats were recorded simultaneously using IoX software (EMKA Technologies Inc.; France), stored in a PC, and later formatted for further analysis by custom MATLAB scripts. Two weeks after recovery from the second surgery, VNS protocol was initiated at W7 and continued for 8 weeks (Figure 1A). A wireless pulse generator was connected to each rat by the experimenter and programmed using the software for applying VNS in awake behaving rats. VNS was delivered five times a week (on weekdays except holidays) in the morning (around 10 AM) based on fixed parameters: constant-current, biphasic, and charge-balanced square-wave pulses (1 mA/phase, 100 μs/phase) at 30 Hz applied for 2 min ON/2 min OFF for 10 min (Figure 1C). To provide Sham animals with similar environmental stimuli, cage lids were removed and the same amount of animal handling (except attaching stimulator) was performed by experimenters. After attaching the wireless pulse generator to the connectors (Figure 1C), VNS was scheduled by the experimenter and Treatment rats received VNS simultaneously while moving freely inside the cage.

### Data Analysis

#### Cardiovascular System Assessment

Cardiovascular effects of VNS were studied using a thorough analysis of continuous ECG signals acquired by the data acquisition system. LabChart Pro software (ADInstruments; CO, United States) was first used for beat-to-beat waveform detection based on predefined parameters for rat ECG. Then, abnormal beats were excluded based on the estimated heart rate less than 250 bpm or more than 500 bpm, and ECG morphology and HRV analyses were performed using custom scripts in MATLAB (Mathworks, Inc.; MA, United States). Since implantation of the cuff electrode around the cervical vagus nerve does not significantly affect the cardiovascular system and behavior in rats (Yaghouby et al., 2019), ECG parameters on the week before the experiment started (W6) represented as baseline. ECG features including morphology and HRV parameters were estimated from 10-min non-overlapping ECG segments. To characterize the morphology of the ECG, the time between P-wave onset to QRS onset (PR) was estimated. HRV analyses using time-domain, frequency-domain, and non-linear techniques were also performed to characterize the autoregulation of heart rhythmicity by the autonomic nervous system. As time-domain HRV, we calculated the average R–R interval (RR) and square root of the mean squared differences of successive R–R intervals (RMSSD). For frequency-domain analysis, the absolute power of different frequency bands commonly used in rats including low-frequency (LF power: 0.2–0.75 Hz) and high-frequency (HF power: 0.75–2.5 Hz) were first estimated using Lomb–Scargle periodogram estimation of the R–R interval time series and the proportion of HF power [HF/(LF + HF)] and power ratio (LF/HF) were calculated. For HRV non-linear analysis, Poincaré plot and detrended fluctuation analysis (DFA) were selected to reflect short-term and long-term variabilities of fluctuations in the R–R interval time series (Shaffer and Ginsberg, 2017). Specifically, the ratio of standard deviation perpendicular to the line of identity (SD1) over the standard deviation parallel to the line of identity (SD2) in the Poincaré plot and the ratio of short (Alpha1) over long-term (Alpha2) fractal exponents of the DFA were calculated as non-linear HRV features. To fully characterize the cardiovascular changes, cardiovascular parameters were calculated and compared between groups (Treatment vs Sham and male vs female) to evaluate short-term and long-term VNS effects. To study short-term effects, features only from eight weeks of VNS (W7–14) were selected and each feature (estimated from 10-min ECG segments) was compared between pre-VNS and post-VNS segments across groups. To study long-term effects, the average of features during the post-VNS period (the first 60 min after VNS), were tracked as week-by-week trends started from W7 onward. To avoid the confounding effect of animal handling and stress during VNS sessions, pre-VNS features for statistical analysis were estimated from 10-min ECG segments centered 30 min before each VNS session.

#### Immune System Assessment

Inflammatory responses following VNS were assessed by measuring plasma levels of TNF-α, IL-1β, IL-6, and IFN-γ cytokines from systemic blood samples.

#### Statistical Analysis

All results are presented as mean ± standard error of the mean (SEM), unless otherwise indicated in the text. Weekly cardiovascular parameters for each animal were averaged across five independent VNS sessions after extreme outliers were excluded based on Tukey’s hinges (below first quartile −3 × interquartile range (IQR) and above third quartile +3 × IQR). Analysis of variance (two-way ANOVA) with multiple comparisons and *post hoc* analysis with Bonferroni’s test was used to perform statistical comparisons between Treatment and Sham groups at different timepoints post-VNS. We also compared timepoints within groups using two-way ANOVA followed by Tukey’s multiple-comparison test. However, to enhance readability, we only marked significant results for between-group effects over time in figures. For comparing sex effects, the first 1 h of data post-VNS (i.e., 0 to +50 min data points) was compared between male and female rats from the Treatment and Sham groups using the two-sample *t*-test. Values of *p* < *0.01* and *p* < *0.05* were considered statistically significant (*p*-values corrected for repeated comparisons). Statistical analysis was made in Prism 8 (GraphPad Software, CA, United States).

## Results

### Cardiovascular Effects of VNS

**FIGURE 2:**
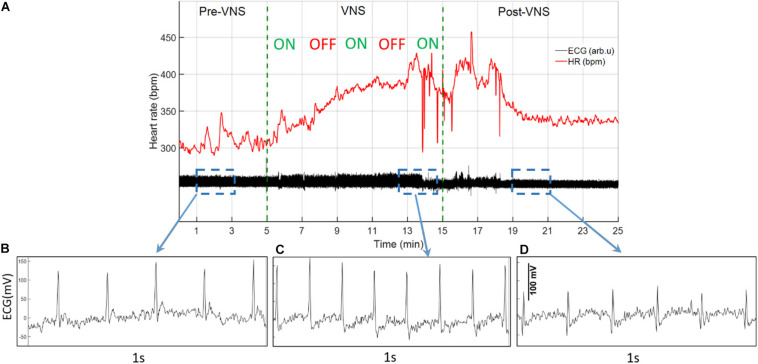
A sample ECG recording and estimated instantaneous heart rate (HR) from a male rat during VNS. **(A)** ECG signal and estimated HR before, during, and after a VNS session. **(B–D)** 1s ECG snippet for baseline (Pre-VNS), stimulation (VNS), and recovery (Post-VNS), respectively, indicating cardiovascular changes associated with the VNS.

ECG signals recorded from 16 rats (*n* = 8 in Treatment and n = 8 in Sham group) were analyzed for total duration of the experiment to characterize cardiovascular effects of VNS and potential sex differences. For the Sham group, only two female rats were available with continuous ECG signals from W6 onward because of early battery failure in implanted wireless ECG monitoring devices. Figure 2A demonstrates an ECG signal and instantaneous heart rate (HR) from a male rat during a VNS session. Figures 2B–D present 1s ECG snippets for Pre-VNS, VNS and Post-VNS, respectively. As can be seen, HR elevates during VNS by more than 30% and then recovers shortly to a level slightly higher than the pre-VNS period. A similar cardiac effect was previously observed in a preliminary cohort of female rats using comparable VNS parameters (Yaghouby et al., 2019). To correct for inter- and intra-subject variabilities in pre-VNS cardiovascular features (shown in Table 1), estimated features from each animal were normalized by dividing by the pre-VNS values (i.e., estimated features from an ECG segment centered at 30 min before VNS onset). To study long-term effects as week-by-week trends, the average of ECG features during the post-VNS period (the first 60 min after VNS) were normalized by dividing by the corresponding timepoints from the week before VNS started or baseline week (W6). Finally, normalized features were tracked for VNS (W7–14) and recovery (W15–18) weeks. Sex difference comparisons for long-term effects were performed by averaging features in three phases: *VNS_Early* (W7–10), *VNS_Late* (W11–14), and *Recovery* (W15–18).

**TABLE 1:** Baseline ECG features estimated from the pre-VNS timepoint in different groups (mean ± SEM).

	Sham (*n* = 6)		Treatment (*n* = 8)	
	Male (*n* = 4)	Female (*n* = 2)	Male (*n* = 4)	Female (*n* = 4)
PR (ms)	46.70 ± 0.49	42.23 ± 0.73	45.75 ± 0.81	44.45 ± 1.20
RR (ms)	193.41 ± 7.15	177.65 ± 1.00	184.23 ± 3.12	180.84 ± 1.86
RMSSD (ms)	17.43 ± 8.10	9.00 ± 4.27	7.96 ± 2.41	10.51 ± 3.94
HF/(LF + HF)	0.54 ± 0.11	0.52 ± 0.12	0.50 ± 0.07	0.48 ± 0.06
LF/HF	1.29 ± 0.54	1.1 ± 0.54	1.07 ± 0.26	1.2 ± 0.21
SD1/SD2	0.51 ± 0.19	0.39 ± 0.16	0.36 ± 0.08	0.38 ± 0.1
Alpha1/Alpha2	1.1 ± 0.05	0.93 ± 0.09	0.98 ± 0.07	0.92 ± 0.02

*PR, time between P-wave onset to QRS onset in ECG; RR, time interval between consecutive R-waves in ECG; RMSSD, square root of the mean squared differences of successive R–R intervals; HF/(LF + HF), proportion of high-frequency (0.75–2.5 Hz) power from R–R interval time series; LF/HF, low-frequency (0.2–0.75 Hz) to high-frequency (0.75–2.5 Hz) power ratio; SD1/SD2, the ratio of the standard deviation perpendicular to the line of identity (SD1) over the standard deviation parallel the line of identity (SD2) in Poincaré plot; Alpha1/Alpha2, the ratio of short (Alpha1) over long-term (Alpha2) fractal exponents of the detrended fluctuation analysis (DFA).*

#### ECG Morphology and HRV Time-Domain Features

**FIGURE 3:**
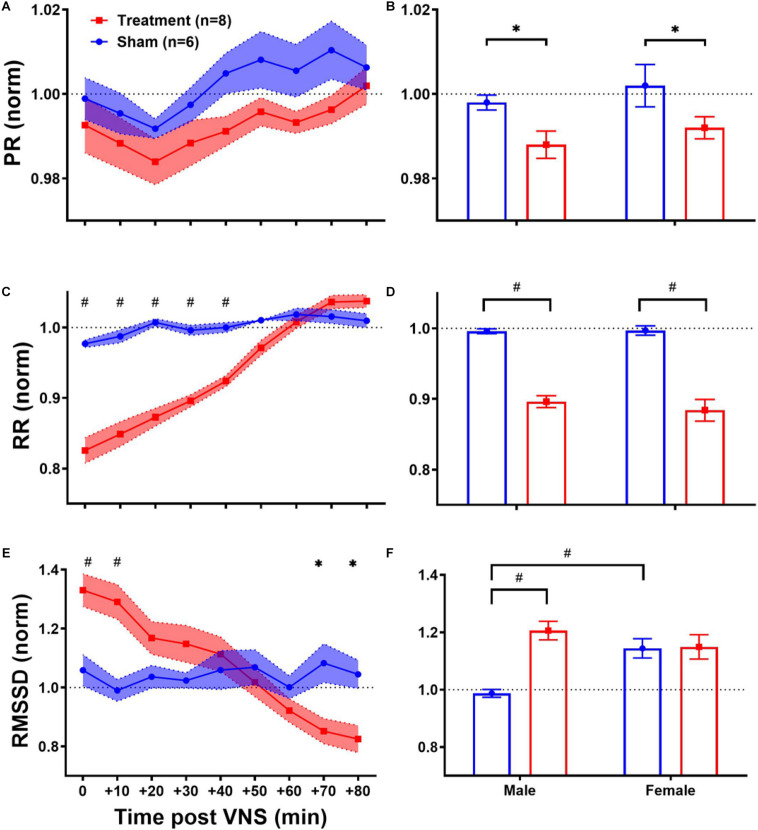
Normalized short-term trends for ECG morphology and HRV time-domain features (mean ± SEM). **(A,B)** PR reduces in Sham and Treatment rats, and the effect was more pronounced in the Treatment group with longer recovery to the baseline level (*p* > 0.05). The average effects for the first hour post-VNS (from 0 to + 50min) shows significant differences between Sham and Treatment groups for male and female rats (*p* < 0.05) without any significant sex differences. **(C,D)** RR significantly reduces in the Treatment group with almost one-hour recovery time to the baseline level. The average effects for the first 1 h post-VNS (from 0 to +50 min) show significant differences between Sham and Treatment groups without any significant sex differences. **(E,F)** RMSSD significantly increases by ∼30% in the Treatment group with ∼20 min recovery. The average trend shows significant treatment effect only in male and significant sex difference in Sham groups. *p < 0.05 and ^#^*p* < 0.01.

As shown in Table 1, ECG morphology and HRV time-domain features (PR, RR, and RMSSD) during baseline were not significantly different between male and female rats from each group (Treatment vs Sham), although inter-subject variability was notably reflected as relatively large variance particularly for RMSSD. Figure 3 summarizes short-term trends for ECG morphology and HRV time-domain features normalized to the pre-VNS period for each group. Significant differences were found by two-way ANOVA for RR and RMSSD between Treatment and Sham animals when male and female rats were pooled together (Figures 3C,E, *p* < 0.05 or 0.01). The effect of VNS during the first hour was significant in both male and female rats for PR and RR and only in male rats for RMSSD (Figures 3B,D,F, *p* < 0.05 or 0.01). PR, indicating the conduction velocity of cardiac impulse, significantly reduced in male and female groups (Figure 3B, *p* < 0.05) and then slowly recovered to the baseline level after about one hour (Figure 3A). While no obvious trend was observed in the Sham group, significant and prolonged suppression in RR and short-term variability (reflected as elevated RMSSD) were apparent in the Treatment group (Figures 3C,E; *p* < 0.05 or 0.01). VNS seemed to equally affect post-VNS RR values in male and female rats as shown in Figure 3D (*p* < 0.01). Compared to the Sham group, RMSSD significantly increased by about 30% in the Treatment group, but the recovery was faster than RR (Figure 3E). The sex-specific VNS effect on RMSSD was obvious where a significant increase was observed only in male rats (Figure 3F; *p* < 0.01). In general, male and female rats responded to VNS similarly and no sex difference was observed except for RMSSD.

**FIGURE 4:**
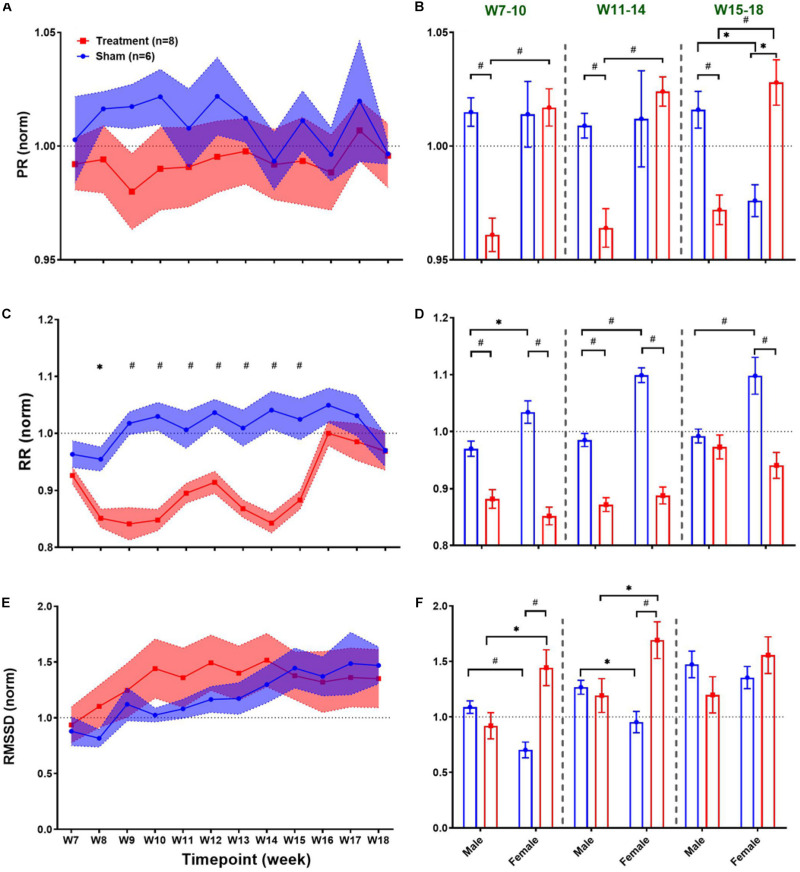
Normalized long-term trends for ECG morphology and HRV time-domain features (mean ± SEM). **(A,B)** PR slightly reduces in the Treatment group and increases in the Sham group (not significant; *p* > 0.05) and slowly recovers toward the VNS endpoint (W14). The average effects for *VNS_Early* (W7–10), *VNS_Late* (W11–14), and *Recovery* (W15–18) periods are compared between groups (Treatment vs Sham and male vs female). Male–female differences are shown for three periods. **(C,D)** The long-term effect of VNS on RR is significantly different between the Treatment and Sham groups even during the first week of recovery (W15). While sex difference was significant only in the Sham group over three phases, Treatment effect remains significant until W15 for both male and female rats and only for female rats after W15. **(E,F)** There is no significant difference between the Treatment and Sham groups in the long-term effect of VNS on RMSSD. However, when male and female rats were compared from each group, a significant increase in RMSSD was observed in female rats by VNS, during *VNS_Early* and *VNS_Late* phases. *p < 0.05 and ^#^*p* < 0.01.

Long-term effects of VNS on cardiovascular variables can be studied to evaluate possible adaptation of cardiac activity over successive stimuli during several weeks of the experiment. Weekly trends for the post-VNS period (the first 60 min after VNS) in ECG morphology and HRV time-domain features are shown in Figure 4. As we observed in short-term trends in Figure 3, the first 60-min post-VNS incorporates immediate and sometimes significant cardiovascular effects; hence, this period has been used to assess possible long-term effects. The left panel of Figure 4 demonstrates post-VNS weekly trends (normalized to the baseline or W6) for PR, RR, and RMSSD features, where only a significant difference was found between Treatment and Sham groups for RR (Figure 4C; *p* < 0.05 or 0.01). When male and female rats from each group were compared in long-term effects separately (right panel of Figure 4), significant differences were found during *VNS_Early* (W7–10), *VNS_Late* (W11–14), and *Recovery* (W15–18) phases (Figures 4B,D,F; *p* < 0.05 or 0.01). While there seems to be negligible long-term effects for PR in the Treatment group (Figure 4A), significant sex differences were found during three long-term phases shown in Figure 4B where PR suppression was observed only in male rats when VNS was ON (*p* < 0.01). VNS significantly reduced RR in the Treatment group (Figure 4C), and the effect was similar for male and female rats during the *VNS_Early* (W7–10) and *VNS_Late* (W11–14) phases (Figure 4D). However, during *Recovery* when VNS was OFF (W15–18), the effect remained significant only for the female group (Figure 4D, *p* < 0.01), showing that the effect of VNS on RR for female lasted longer even after VNS was turned off. Interestingly, male and female rats from the Sham group showed significantly different RR values during the three phases shown in Figure 4D (*p* < 0.05 or 0.01). While there was no significant difference between Treatment and Sham groups for long-term RMSSD trends (Figure 4E), further analysis during three long-term phases showed significant effects of VNS only in female rats (Figure 4F; *p* < 0.05). This analysis also showed that when male and female rats were compared as shown in Figure 4B, there are significant differences even during *Recovery* phase (*p* < 0.01 or 0.05).

#### HRV Frequency-Domain Features

**FIGURE 5:**
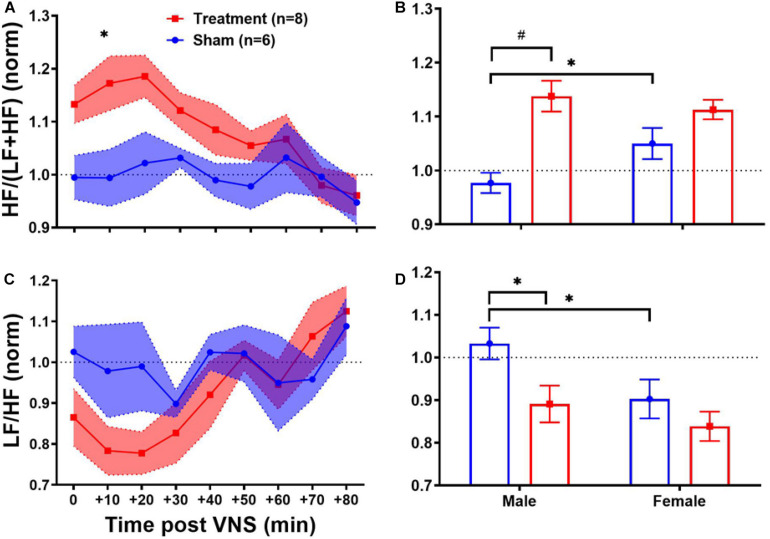
Normalized short-term trends for HRV frequency-domain features (mean ± SEM). **(A,B)** HF/(LF + HF) indicating the HF proportion of total power shows significant increase in the VNS group with relatively slow transition to the baseline value. Male–female comparison shows that the effect is only significant in male rats. **(C,D)** LF/HF power ratio or sympathovagal balance shows reduction by VNS (not significant) and slow recovery indicating the predominance of parasympathetic modulation. Male–female comparison shows that the effect is only significant in male rats. *p < 0.05 and ^#^*p* < 0.01.

Heart rate variability frequency-domain analysis was performed by power spectral analysis of the R–R interval time series. As shown in Table 1, no significant difference was found between pre-VNS values of frequency-domain features in male and female rats. Figure 5 summarizes normalized short-term trends for the HRV frequency-domain features for each group. A significant difference between Treatment and Sham groups was only found for HF/(LF + HF) (Figure 5A, *p* < 0.05). However, the effect of VNS on HF/(LF + HF) during the first hour was only significant in male rats (Figure 5B, *p* < 0.01). Compared to the Sham group, HF/(LF + HF) significantly increased by about 20% in the Treatment group and recovery took about 1 h (Figure 5A, *p* < 0.05), and the effect was significant and pronounced only in the male group (Figure 5B, *p* < 0.01). Trends for LF/HF almost mirrored HF/(LF + HF) but without significant differences between the Treatment and Sham groups (Figure 5C). However, when the post-VNS LF/HF values were compared independently in male and female rats, significant suppression only in male rats was observed (Figure 5D; *p* < 0.05). Overall increase in HF/(LF + HF) and suppression in LF/HF observed in male rats indicated that sex-specific parasympathetic dominance lasted for a relatively long time and recovery to baseline level was evident after about 60–90 min (Figure 5).

**FIGURE 6:**
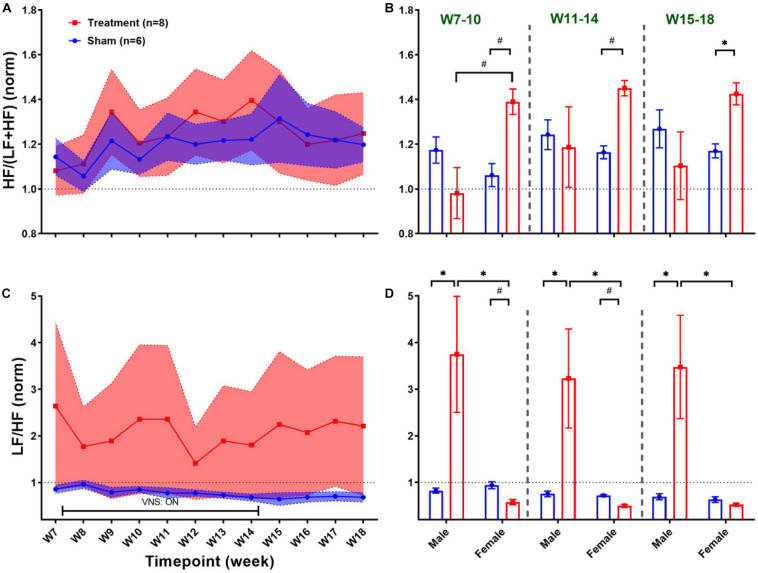
Normalized long-term trends for HRV frequency-domain features (mean ± SEM). **(A,B)** The long-term effect of VNS on HF/(LF + HF) is not significantly different between the Treatment and Sham groups. However, when male and female rats were compared from each group, a significant increase in HF/(LF + HF) was observed in female rats by VNS, during *VNS_Early*, *VNS_Late*, and *Recovery* phases. **(C,D)** There is no significant difference between the Treatment and Sham groups in long-term effect of VNS on LF/HF. However, when male and female rats were compared from each group, a significant increase in male and reduction in female rats was observed by VNS during all three shown phases. *p < 0.05 and ^#^*p* < 0.01.

Long-term effects of VNS on frequency-domain HRV features are summarized in Figure 6. The left panel of Figure 6 demonstrates post-VNS weekly trends (normalized to the baseline or W6) for HF/(LF + HF) and LF/HF features. No significant differences between the Treatment and Sham groups were observed and the variability appears to be larger in the Treatment group. When male and female rats from each group were separately compared for long-term effects (right panel of Figure 6), significant differences were found during *VNS_Early* (W7–10), *VNS_Late* (W11–14), and *Recovery* (W15–18) phases (Figures 6B,D; *p* < 0.05 or 0.01). While long-term effect of VNS on HF/(LF + HF) seems to be different in male and female rats during *VNS_Early* and *VNS_Late* phases (reduction in male and increase in female), the difference was only significant during the *VNS_Early* phase (Figure 6B; *p* < 0.01). For HF/(LF + HF), the effect of VNS on the female group remains significant during the *VNS_Late* and even *Recovery* phases (Figure 6B; *p* < 0.05 or 0.01). Although no significant effect on LF/HF was found when the Treatment group was compared to the Sham (Figure 6C), male–female comparison revealed several significant differences during the *VNS_Early*, *VNS_Late*, and *Recovery* phases (Figure 6D; *p* < 0.05 or 0.01). Particularly, female rats from the Treatment group showed significantly lower LF/HF than male rats from the Treatment group during all the three shown phases. This analysis also showed that when male and female rats were compared independently, there were some significant differences even in the Sham group (*p* < 0.05 or 0.01).

#### HRV Non-linear Features

**FIGURE 7:**
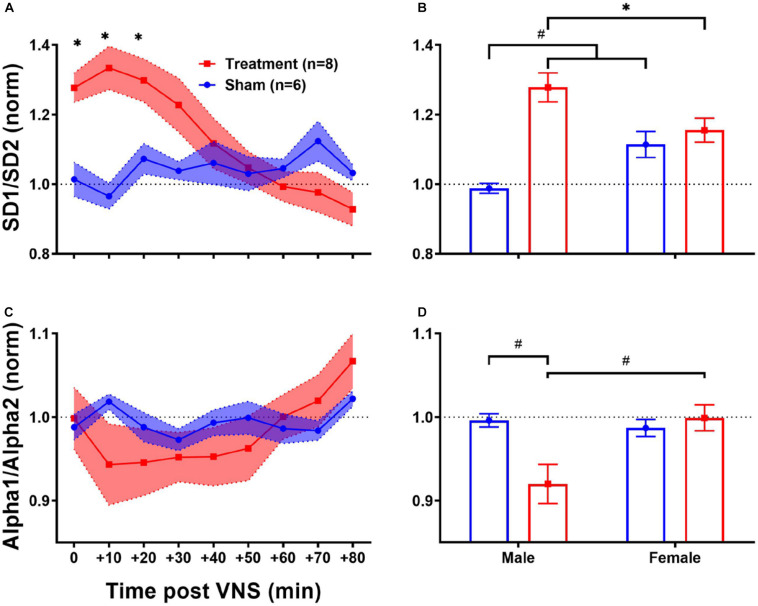
Normalized short-term trends for HRV non-linear features (mean ± SEM). **(A,B)** SD1/SD2 significantly increases by VNS and then recovers after about 30–40 min. Male–female comparison revealed that the effect was only significant in male rats. **(C,D)** Alpha1/Alpha2 slightly decreases by VNS without significant difference compared to the Sham group. Male–female comparison shows that the effect is only significant in male rats. *p < 0.05 and ^#^*p* < 0.01.

Since the regulatory role of the autonomic nervous system on cardiovascular function is known to be through a non-linear physiological mechanism (Shaffer and Ginsberg, 2017), complex non-linear dynamics of the heart affected by VNS can be further quantified using HRV non-linear features. As shown in Table 1, HRV non-linear features (SD1/SD2 and Alpha1/Alpha2) pre-VNS were not significantly different between male and female rats from each group (Treatment vs Sham). Figure 7 summarizes short-term trends for HRV non-linear features normalized to the pre-VNS period for each group. Significant differences between Treatment and Sham animals when male and female are grouped were only found for SD1/SD2 (Figure 7A, *p* < 0.05). However, the effect of VNS on SD1/SD2 during the first hour was only significant in male rats (Figure 7B, *p* < 0.01). While slight and non-significant reduction was observed for Alpha1/Alpha2 in the Treatment group compared with Sham (Figure 7C), this reduction was only significant in male rats (Figure 7D; *p* < 0.01). An overall increase in SD1/SD2 and suppression in Alpha1/Alpha2 was observed only in male rats indicating sex differences in non-linear analysis of HRV by VNS (Figure 7).

**FIGURE 8:**
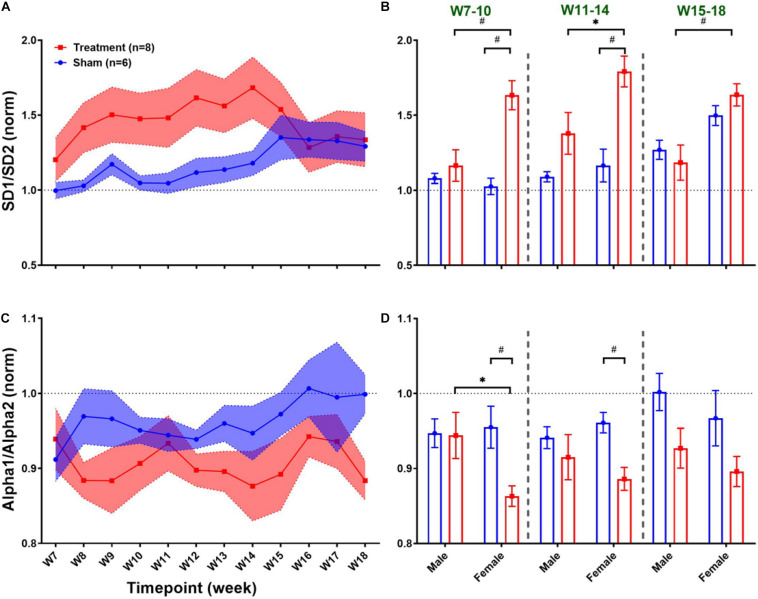
Normalized long-term trends for HRV non-linear features (mean ± SEM). **(A,B)** The long-term effect of VNS on SD1/SD2 is not significantly different between Treatment and Sham groups; however, an increasing trend during VNS is obvious in the Treatment group. VNS effects on SD1/SD2 seem to be similar in male and female rats, but a significant effect was only observed in the female group for early VNS and late VNS phases. **(C,D)** While VNS slightly decreases Alpha1/Alpha2, there is no significant difference between Treatment and Sham groups in the long-term effect. However, when male and female rats were compared from each group, this reduction appears to be significant only in female rats during *VNS_Early* and *VNS_Late* phases. *p < 0.05 and ^#^*p* < 0.01.

Long-term effects of VNS on non-linear HRV features are summarized in Figure 8. The left panel of Figure 8 demonstrates post-VNS weekly trends (normalized to the baseline or W6) for SD1/SD2 and Alpha1/Alpha2 features. No significant difference between the Treatment and Sham groups was observed while the effect seems noticeable on SD1/SD2. When male and female rats from each group were compared in long-term effects (right panel of Figure 8), significant differences were found only in female rats particularly during *VNS_Early* (W7–10) and *VNS_Late* (W11–14) phases for SD1/SD2 and Alpha1/Alpha2 (Figures 8B,D; *p* < 0.05 or 0.01). The long-term effects of VNS on SD1/SD2 and Alpha1/Alpha2 were similar between male and female rats (increase in SD1/SD2 and decrease in Alpha1/Alpha2) during the *VNS_Early* and *VNS_Late* phases (Figures 8B,D; *p* < 0.05 or 0.01). However, the effect was significant for SD1/SD2 during the three long-term phases shown in Figure 8B (*p* < 0.05 or 0.01).

### Inflammatory Effects of VNS

Concentration of four plasma cytokines during the baseline (W1) are shown in Table 2 for male and female rats from Treatment and Sham groups. As can be seen, baseline concentration of plasma cytokines does not significantly differ between male and female rats, although the variability between animals appear to be relatively large (Table 2).

**TABLE 2:** Baseline plasma cytokine levels.

	Sham (*n* = 8)		Treatment (*n* = 8)	
	Male (*n* = 4)	Female (*n* = 4)	Male (*n* = 4)	Female (*n* = 4)
TNF-α (pg/mL)	6.59 ± 1.49	7.5 ± 2.72	4.01 ± 0.53	5.24 ± 0.66
IFN-γ (pg/mL)	85.72 ± 35.49	108.39 ± 48.42	34.07 ± 6.53	48.17 ± 15.97
IL-1β (pg/mL)	9.09 ± 7.15	13.21 ± 3.27	5.88 ± 1.08	10.38 ± 3.86
IL-6 (pg/mL)	87.98 ± 37.28	87.39 ± 27.93	94.23 ± 6.98	68.11 ± 27.26

**FIGURE 9:**
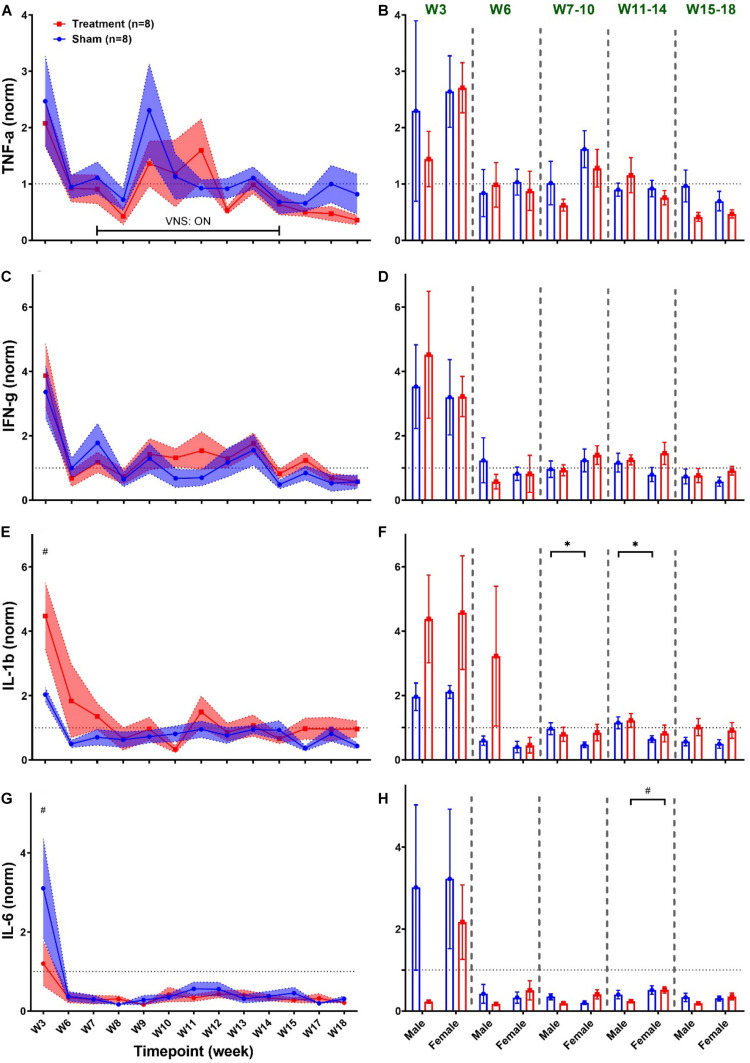
Weekly concentration of plasma cytokines normalized to the baseline during different experimental timepoints (mean ± SEM). **(A,B)** TNF-α increases by almost four-fold in both Treatment and Sham groups after implantation of the ECG device on W3 with no consistent trend or significant change by the VNS. Male–female comparison does not reveal any significant effects or sex differences during the five independent phases. **(C,D)** IFN-γ elevates by the VNS in all rats and then quickly recovers and remains unchanged without significant differences between the Treatment and Sham groups. Male–female comparison does not reveal any significant effects or sex differences during the five independent phases **(E,F)** IL-1β significantly increases in the Treatment group after the first surgery and then recovers within two weeks and remains unchanged without any difference compared to the Sham group. Male–female difference was only found in the Sham group during the *VNS_Early* and *VNS_Late* phases. **(G,H)** IL-6 significantly increases in the Sham group after the first surgery and then sharply falls below the baseline level at a comparable range as Treatment group with no significant difference. Male–female comparison reveals significant difference in Treatment during the *VNS_Late* phase. *p < 0.05 and ^#^*p* < 0.01.

Figure 9 summarizes weekly concentration levels of four plasma cytokines from male and female rats in each group. The timeline for cytokine measures was slightly different from the one for cardiovascular parameters. First, we collected blood samples and assessed inflammatory cytokines in multiple weeks before VNS (W1, W3, and W6) to characterize the effect of surgeries. Also, for post-VNS recovery, we have three timepoints available including W15, W17, and W18. Cytokine concentrations shown in Figure 9 were normalized to the value from the baseline (i.e., W1) for each rat, and weekly trends for the Treatment and Sham groups are shown on the left panel. As can be seen, there is a surge in concentration of cytokines in both groups right after implantation (on W3), which is significantly different between the Treatment and Sham groups for IL-1β and IL-6 (Figures 9E,G; *p* < 0.01). Apart from the surgery effect, there does not appear to be any effects by the VNS. Male–female comparison has been done in five different experimental phases: Post-Surgery1 (W3), Post-Surgery2 (W6), *VNS_Early* (W7–10), *VNS_Late* (W11–14), and *Recovery* (W15–18) phases. As shown on the right panel of Figure 9, apart from the effect of the first surgery on W3, there was no predominant effect by the VNS on males and females. However, there was a significant sex difference in IL-1β from Sham group during *VNS_Early* and *VNS_Late* phases, which might be due to variability in subjects or measurement error (Figure 9F; *p* < 0.05). The only significant and notable difference that we observed for cytokine concentration by VNS was in the *VNS_Late* phase of IL-6 where the concentration of this cytokine was significantly different between male and female rats from the Treatment group (Figure 9H; *p* < 0.01). Overall, the concentration of all plasma cytokines shown in Figure 9 increased notably after the first surgical implantation on W3. Apart from the initial surge at W3, there do not seem to be any noteworthy changes by VNS on shown cytokines. A slight reduction in plasma IL-6 expression by VNS was observed only in male rats during the *VNS_Early* phase (Figure 9H). However, there was neither consistent nor significant effect by the VNS or sex differences in other observed trends.

## Discussion

One of the challenges in the development of medical device technologies is contribution of sex differences in variations observed during assessment of safety and effectiveness (Dhruva and Redberg, 2012). Considering sex differences in design and implementation of preclinical experiments may provide more translational insights on development of medical devices. To overcome technical challenges related to the implementation of long-term VNS experiments in awake behaving rodents, we developed a robust animal model using rats (Yaghouby et al., 2019). In the current study, we evaluated the potential role of sex differences in cardiovascular and inflammatory responses to long-term VNS using continuous ECG monitoring and weekly blood sample analysis using this animal model. To our knowledge, this experimental study is the first attempt to systematically assess the sex-specific changes by VNS on cardiovascular and immune systems.

Current experimental and clinical evidence from literature supports that VNS through extensive innervation with SA and AV nodes can affect HR and HRV (Shen and Zipes, 2014; Kapa et al., 2016). From a clinical perspective, VNS-induced cardiac changes in epilepsy patients have been reported to be either rare or insignificant. However, activation of the brainstem through afferent pathways can variably alter both vagal and sympathetic cardiovascular modulations without significant hemodynamic effects in epilepsy (Garamendi et al., 2017). Nevertheless, cardiovascular effects of VNS using animal models have shown significant variability in response depending on the factors such as selected stimulation parameters and target nerve, as well as experimental condition (Ardell et al., 2017).

Although sex differences in autonomic regulation of cardiovascular and hemodynamic systems have been widely documented (Chandler and Dicarlo, 1998; Reckelhoff, 2001), differences in heart innervation and morphologic and morphometric alterations in the vagus nerve were only studied in an experiment using spontaneously hypertensive rats and did not reveal dissimilarities between male and females (Licursi De Alcantara et al., 2008). Sex differences in VNS cardiovascular effects may originate from neurohormonal differences or inconsistency in the sensitivity of certain nuclei along the cardiac branch pathway in male and female subjects (De Couck et al., 2017). While clinical evidence from healthy men and women revealed that sex difference should be taken into account when designing HRV studies (Koenig and Thayer, 2016), contribution of sex differences on cardiac off-target effects reflected as HRV when VNS is in play is yet to be fully understood. As one of rare efforts in this context, a clinical study using non-invasive VNS (transcutaneous VNS or t-VNS) showed that compared to men, SDNN was significantly larger in women after VNS (De Couck et al., 2017).

### Short-Term Cardiovascular Effects

Transitional effects of VNS on the cardiovascular system reflected as elevated vagal tone in HRV have been previously reported in experimental studies using rodent models (Chapleau et al., 2016; Lee et al., 2018; Yaghouby et al., 2019). In this study, in addition to an ECG morphology feature (PR), we performed both linear (time and frequency domain analyses) and non-linear techniques to thoroughly evaluate the effects of VNS on cardiovascular variables. Short-term effects of VNS on cardiovascular variables were observed in Treatment rats with approximately 30–60 min recovery time to rebound to the baseline level. However, as shown in Figures 3–8, significant differences between Treatment and Sham groups were observed in selected features from each category including RR, RMSSD, HF/(LF + HF), and SD1/SD2. However, when male and female rats were compared separately between Treatment and Sham groups, significant effects of VNS were observed in some other cardiovascular variables. To eliminate the effects of human interaction on cardiovascular system at resting condition, pre-VNS features were estimated from ECG signals recorded 30 min before the VNS onset (i.e., prior to the presence of experimenters in the room). In addition, to alleviate the large variability observed in cardiovascular parameters (shown in Table 1), estimated features from each rat were normalized with respect to the pre-VNS values. When male and female rats were compared separately, abnormal cardiac rhythms due to premature excitation of the cardiac myocytes by VNS were reflected as relatively shorter PR (Figure 3A). However, this effect was not significant when male and female rats from Treatment groups were compared (Figure 3A). In one comparable effort, VNS did not affect the PR (slightly reduced but not significant) from ECGs recorded in anesthetized rat models of myocardial infarction (Xie et al., 2014). In general, VNS-associated arrhythmias like different degrees of AV block have been reported in epilepsy patients implanted with VNS (Kato et al., 2018). Although the evoked cardiac response to VNS is presumed to involve parasympathetic efferent nerves to the heart, there also exists another parasympathetic pathway mediated by the afferent nerve in central nervous system (Ardell et al., 2017). Cervical VNS can modulate efferent and afferent nerve fibers, and the proportion of those is different between left and right cervical vagus nerve. More than eighty percent of the left cervical branch of the vagus nerve consist of afferent sensory fibers projecting into the medulla in the central nervous system (Berthoud and Neuhuber, 2000). In summary, the cardiovascular effect might vary depending on variables such as stimulation parameters (not only intensity but also frequency, pulse width, and duration), animal condition (awake or anesthetized, healthy or diseased, etc.), the target nerve (left or right cervical nerve), and the contribution of other confounders such as human interaction (Ardell et al., 2017; Yaghouby et al., 2019). Hence, the cardiac response to VNS involves complex interaction between afferent and effects nerve fibers depending on frequency–amplitude–pulse width of electrical stimulation.

HRV time-domain analysis showed a 15–20% decrease in RR with about 1 h recovery time in the Treatment group compared to the Sham group (Figure 3C), confirming our finding using a preliminary cohort of female rats and similar VNS parameters (Yaghouby et al., 2019). This effect was also comparable between male and female rats (Figure 3D). Also, according to Ardell et al. (2017), our stimulation parameters approximately fall around the “HR Augmentation Zone” whereas 10–20% increase in HR is anticipated in awake behaving rats. We used constant-current stimulation pulses to account for variability in nerve impedance and our chosen parameters (i.e., intensity, pulse width, and frequency) have been widely used by other investigators using rat models of VNS (Hulsey et al., 2017). Vagally mediated changes following VNS were reflected by RMSSD, as one of the primary features of time-domain HRV (Figure 3E). Although VNS lengthened RMSSD by more than 20% in the Treatment group, the effect was significantly different from the Sham group during the first 20 min post-VNS (Figure 3E). A notable sex difference was observed where RMSSD increase by VNS was only significant in male rats (Figure 3F; *p* < 0.01). Because of large variability between animals, RMSSD was found to be significantly different between male and female rats from the Sham group, and this appears to be one of the reasons that the VNS effect was not significant in female rats.

Chronic VNS on epilepsy patients through mainly afferent nerve fibers in the left cervical nerve has been shown to suppress sympathetic tone, as measured by skin sympathetic nerve activity (Yuan et al., 2017). The suggested pathway for VNS autonomic changes incorporates activation of the NTS which could either 1) inhibit the sympathetic tone through activation of the caudal ventrolateral medulla which inhibits the rostral ventrolateral medulla or 2) activate the parasympathetic tone through activation of the dorsal motor nucleus of the vagus and the nucleus ambiguous (Clancy et al., 2014). Our observations particularly on HRV frequency-domain analysis (Figure 5) confirmed this suggested pathway. It is generally accepted that the low-frequency component of the R–R interval (LF power) reflects both sympathetic and parasympathetic modulations of the autonomic nervous system while the high-frequency component (HF power) is more specific to the parasympathetic modulation (Shaffer and Ginsberg, 2017). Hence, significantly elevated parasympathetic activity in the Treatment group compared with Sham shown as HF/(LF + HF) in Figure 5A accompanied by suppressed sympathetic activity shown as reduction in LF/HF was evident. While it is generally accepted that the therapeutic outcome of VNS is through activation of the parasympathetic components of the vagus nerve, the cervical vagus nerve comprises significant sympathetic components and electrical stimulation of the cervical branch may retrogradely activate the stellate ganglion (SG) (Chinda et al., 2016). Our HRV frequency-domain analysis indicated that VNS can change cardiovascular autonomic balance toward parasympathetic/vagal dominance in healthy rats, and it has been shown as increased HF/(LF + HF) for about one hour after stimulation. A similar effect of VNS on parasympathetic activity was also reported elsewhere (Clancy et al., 2014).

For healthy human subjects, it was shown that under baseline conditions, the frequency features of HRV in females are characterized by significantly elevated HF and suppressed LF components (Koenig and Thayer, 2016). Our results from the Sham group confirmed significant differences in HF/(LF + HF) and LF/HF power features in female rats compared to males (Figures 5B,D; *p* < 0.05). Also, the effect of VNS was shown to be only significant in the male group where significant increase in HF/(LF + HF) (Figure 5B; *p* < 0.01) and significant reduction in LF/HF (Figure 5D; *p* < 0.05) were notable. It is worth noting that observed trends in female rats are subjected to a larger variability compared to males because of menstrual cycle that repeats every 4–5 days. Estrous cycle is known to alter HRV variables (Kuo et al., 2010) as well as expression of inflammatory cytokines (Arakawa et al., 2014).

Due to variability in sensitivity to noise and experimental conditions, HRV changes by VNS may not be easily detectable through traditional time-domain or frequency-domain analyses. Hence, non-linear analysis of RR intervals has been implemented to reflect any possible effect that may have been overlooked by physiologically comparable linear features. For example, SD1/SD2 reflecting short-term over long-term fluctuations of R–R interval time series showed significant increase in the Treatment group compared to Sham, but the effect was found to be significant only in male rats (Figures 7A,B). Linear features such as LF/HF or RMSSD that reflect similar physiological effects replicated similar changes only in male rats (Figures 3F, 5D). Recent findings from clinical trials of VNS for heart failure have evidenced that VNS elevates the autonomic cardiovascular balance in favor of parasympathetic dominance reflected as increase in the SD1/SD2 ratio (Libbus et al., 2017). A similar effect was observed in both male and female rats from our study, and the effect was significantly pronounced in the male group (Figures 7A,B). Alpha1/Alpha2 is a ratio of brief over long-term R–R interval fluctuations, and from what is shown in Figures 7C,D, VNS seems to significantly affect only the male group. The ratio (Alpha1/Alpha2) reduced by almost 10% in the male group and the effect lasts for more than 60 min (Figure 7C; *p* > 0.05).

### Long-Term Cardiovascular Effects

The immediate cardiovascular change by VNS reflected as short-term analysis demonstrates that VNS effect is notable or sometimes significant for the first 30 min following VNS and full recovery usually occurs after 60 min. This approximate 1-h recovery time indicated that long-term effects of VNS may require further investigation. Since we have used constant VNS parameters over the course of eight weeks, a physiologic compensatory mechanism adapting the central–peripheral nervous system to the stimulation is likely to be involved (Lee et al., 2018). Therefore, we investigated the long-term effects by comparing trends for cardiovascular variables to assess the residual functional effects occurring after termination of the VNS. Our short-term results indicated that on average cardiovascular variables recover to pre-VNS level almost 60 min after VNS is terminated. One possible explanation for this delayed effect could be contribution of VNS to the phenomenon called cardiac memory in which cardiomyocytes dynamics can be adapted based on the pacing history and response to stimuli (Tolkacheva, 2007).

Compared to short-term trends, there were some contradictory results observed in long-term changes by VNS. For example, while PR significantly reduced in male rats shortly after VNS triggered (Figure 3B), the average of the first 60-min segments post-VNS over *VNS_Early* and *VNS_Late* phases (Figure 4B) did not show significant long-term effects in female rats. Long-term trends for HRV time-domain features revealed more transparent sex differences. Female rats, unlike males, tend to have significantly lower RR even during the *Recovery* phase (W15–18) which means that adaptation may take longer in female rats (Figure 4D). Variations in RR particularly for high-frequency (RMSSD) showed significant increase in female rats during *VNS_Early* and *VNS_Late* phases (Figure 4F) whereas short-term RMSSD trends only reflected significant increase in male rats (Figure 3F).

Significant sex differences were observed for long-term frequency-domain HRV features particularly for HF/(LF + HF) where significant increase in female rats during *VNS_Early*, *VNS_Late*, and even *Recovery* phases were observed reflecting long-term effects of VNS on vagal modulation of cardiac rhythms (Figure 6B). While short-term sympathovagal balance (i.e., LF/HF) significantly shifted toward vagal predominance only in male rats (Figure 5D), long-term trends showed larger variability in the Treatment group, which was further interpreted as significant increase (sympathetic predominance) in male rats and significant decrease (vagal predominance) in female rats (Figures 6C,D). For non-linear HRV features, opposite trends were observed in male and female rats. While short-term effect of VNS on SD1/SD2 and Alpha1/Alpha2 was only significant in male rats, long-term effects were only significant in female rats (Figures 8B,D).

### Inflammatory Effects

Vagus nerve stimulation has been shown to induce an anti-inflammatory effect in animal models of various inflammation diseases (Jin et al., 2017). A minimum suppression of 20–30% in TNF-α, IL-1β, and IL-6 levels in systemic plasma by the VNS has been reported in animal models of clone diseases (Jin et al., 2017). Our results agree with previously published evidence that showed VNS in healthy human subjects does not modulate the systemic inflammatory response (Kox et al., 2015). While statistical analysis of the cytokine levels from the Treatment group revealed significant changes for IL-1β and IL-6 right after the first surgery (Figures 9E,G), sex difference effect was only observed at the *VNS_Late* phase of IL-6 where the level was higher in female rats (Figure 9H). The most notable change in the inflammatory system was after the first implantation surgery where the immune system in all animals responded similarly to the implanted device and surgical procedures. Several reasons might explain the insignificant, absent, or reversed presumed VNS-induced anti-inflammatory reactions. First of all, large variability in female groups (for both Sham and Treatment rats) may come from the estrous cycle in rats that were not taken into account in design of our experiment and known to alter expression of inflammatory cytokines (Arakawa et al., 2014). Based on our experimental design, collection of blood samples occurred on the second session of daily VNS from each week (i.e., Tuesdays) and expression of inflammatory cytokines remained unchanged by the VNS (Figure 9). However, it is noteworthy that VNS could have induced significant changes at a different timepoint or timescale; for instance, toward the end of each week where all five VNS sessions have been applied. Based on our study design, blood samples were collected once a week few hours after the VNS session and we believe that this may result in loss of acute responses of the cytokines immediately following the VNS. We also did not use lipopolysaccharide (LPS) during sample processing that could amplify the overall baseline cytokine level, which has been widely used in other studies.

### Study Limitations

Some limitations of the present study should be taken into consideration. As we observed from the HR trend here and shown in our previous study (Yaghouby et al., 2019), the confounding effect of human interaction on cardiovascular variables cannot be completely avoided using this study design. To overcome wireless charging problems associated with pulse generators used in our experiment, we designed a wearable stimulator (as opposed to an implantable one) which is connected to rats using a mount interface attached to the animal’s back by the experimenter. This human interaction right before VNS may influence HR and interrupt the autoregulation mechanism of cardiac rhythms. Hence, baseline cardiovascular variables were estimated from ECG signals 30-min before the VNS onset. Also, we included the Sham group to assess any possible effects of human interaction on ECG features. Unfortunately, the number of female rats from the Sham group for cardiovascular analysis was 50% less than other groups because of early battery failure for implanted ECG devices. However, since we did not observe significant cardiovascular changes in the Sham group, the risk of statistical misinterpretation due to limited sample size could be negligible. As we discussed, variable estrous cycles in female rats can affect cardiovascular and immune system responses. Although our experimental design did not allow us to synchronize VNS and blood draws to the day of diestrus, cardiovascular/inflammatory effects along with vaginal cytology using estrous-matched females can be done in an independent study to further investigate sex-dependent effects. The cuff electrode integrity of this rat model was previously assessed using impedance tracking and device harvest in female rats (Yaghouby et al., 2019). While the timeline of this study was more or less similar, inclusion of male rats that on average gained three times more weight than females at the end of 18 weeks experiment (65% increase in weight for male and 23% gain weight in female) might affect the durability of electrode and leads. We did not notice any significant change in weekly impedance measurements from male and female animals. However, we observed a few broken leads mostly in male rats at the terminal procedure. Therefore, significant difference in weight gain over the course of chronic experiments should be taken into the account for initial design of electrode and size of leads. Also, it is critical to harvest the electrode at the end of the experiment to investigate possible lead failure.

## Conclusion

The objective of this study was to assess the potential sex differences associated with VNS effects on the cardiovascular and immune systems. This was studied using long-term rodent models implanted with wireless ECG devices and VNS cuff implants. In a cohort of male and female rats, significant differences were observed for HRV analyses in short-term and long-term effects. However, inflammatory biomarker assessment did not reveal significant effects by VNS or notable sex differences.

## Data Availability Statement

The raw data supporting the conclusions of this article will be made available by the authors, without undue reservation.

## Ethics Statement

The animal study was reviewed and approved by FDA’s Institutional Animal Care and Use Committee (IACUC).

## Author Contributions

FY and SV: conceptualization, device design, and surgery. KJ, UH, SA, and FY: experiment and data collection. FY, KJ, and SV: data analysis and manuscript preparation. SV: funding acquisition. All authors contributed to the article and approved the submitted version.

## Conflict of Interest

The authors declare that the research was conducted in the absence of any commercial or financial relationships that could be construed as a potential conflict of interest.
